# Use of the internet by patients attending specialist clinics in Sri Lanka: a cross sectional study

**DOI:** 10.1186/1472-6947-9-12

**Published:** 2009-02-12

**Authors:** Mahinda Kommalage

**Affiliations:** 1 Department of Physiology, Faculty of Medicine, University of Ruhuna, Karapitiya, Galle, Sri Lanka

## Abstract

**Background:**

The internet is a relatively new medium of disseminating health information. Studies on Internet usage for health information are mainly done in developed countries and very few studies have been carried out in developing countries.

**Methods:**

The Internet usage of patients who were attending specialist clinics in Teaching Hospital Karapitiya and Southern Hospital in Galle, Sri Lanka was investigated. The study was carried out on the following specialities; General Medicine, Pediatrics, General Surgery and Cardiothoracic surgery. Information was collected using an investigator-administered questionnaire while patients were waiting for a consultation.

**Results:**

Three hundred and fifty five patients (or guardians in the Pediatric clinic) participated in the study. One hundred seventy two (48.3%) participants have heard about the Internet. There was a relationship between awareness of the Internet and age, educational level and the clinic attended. There was no difference of awareness depending on the gender or the hospital. Only three participants (0.97%) have used the Internet to find information about their disease conditions. Close relatives searched the Internet about the conditions of two participants. Altogether, the Internet was used to search information on the disease condition of five participants (1.4%).

**Conclusion:**

Very low usage of the Internet for health information retrieval in this study is probably due to low awareness of the Internet and low educational level. This low usage of Internet and the associated reasons shown in this study can be generalized to Sri Lanka and probably to other low-income countries that have lower educational level than Sri Lanka.

## Background

The Internet is a relatively new medium of disseminating health information. Availability, cost and accessibility make the Internet more popular than other media. It has been found that over one hundred million people use the Internet for healthcare information worldwide. On any given day, more people go online for medical advice than actually visit health professionals in the world [1].

Previous studies in developed countries have shown that a significant proportion of patients use the Internet to find information related to their condition. Sixty four percent of parents of children at a pediatric hospital in Sydney, Australia [2] and 43% of parents of children at Cincinnati Children's Hospital in USA [3] used the Internet to seek health related information about the conditions of their children. Fifty percent of participants attending a pain centre in Groningen in the Netherlands [4], 43% of patients in a cancer care centre in Edmonton in Canada [5] and 39% of elderly arthritis patients in Virginia in USA [6] used the Internet to seek health information related to their conditions.

Studies on Internet usage for health information have been mainly done in developed countries and very few studies have been carried out in developing countries. The keywords 'Internet health information and UK' generated over 500 results whereas the keywords 'Internet health information' with Bangladesh, Sri Lanka and Nepal generated only one, two and eight results in PubMed, respectively. From those two studies on Sri Lanka, only one study is related to the Internet usage for health information. This study was an analysis of the entries made on the guest book and direct inquiries made to the webmaster on one Sri Lankan medical website [7]. There are two studies on the Internet usage for educating medical professionals in Sri Lanka but not on educating patients [8,9].

However, Internet usage has grown dramatically in developed countries as well as in developing countries during the last 7 years [10]. Therefore, it is reasonable to assume that there is a considerable use of the Internet for health information retrieval in developing countries. Internet usage in Sri Lanka has increased in the last few years very significantly [11]. Health status and the literacy rate are higher in Sri Lanka compared to other low-income countries [12]. Therefore, we assumed that there was considerable usage of the Internet by patients in Sri Lanka. This study was designed to investigate the usage of the Internet to retrieve health information by a group of patients in Galle, Sri Lanka.

## Methods

Internet usage to find information about their disease conditions by patients who were attending specialist clinics, in a government tertiary care hospital, and a private hospital in Galle, were investigated. The two hospitals are Teaching Hospital Karapitiya (THK), which is the main government hospital in Southern province of Sri Lanka, and Southern Hospital (SH), which is a private hospital in Galle, Sri Lanka.

The study was carried out in the clinics of the following specialities: General Medicine, Pediatrics, General Surgery and Cardiothoracic Surgery. A fixed appointment system with a specific time for each patient is not practiced in these two hospitals, which lead to a significant waiting time for the patients prior to the consultation. Every other patient was interviewed using an investigator-administered questionnaire while they were waiting for the consultation.

The questionnaire was designed to collect the following information: age, sex, educational level, area of residence, reason for attending the clinic, whether they have heard about the Internet (awareness about the Internet), whether patient or somebody else has searched the Internet regarding his or her condition. Face validation was done for the questionnaire, which was in Sinhala language. The study was carried out for four days in each hospital between 10th June and 28th June 2008. Ethical approval was obtained from the Ethical Review Committee of the Faculty of Medicine, University of Ruhuna, Sri Lanka (Reference – 27/07/2007).

T-test and Chi-Square test were used to compare the awareness about the Internet with age, educational level and the clinic they were attending. Logistic regression analysis was done for these variables. Results were analyzed using SPSS software version 15.0.

## Results

Three hundred and fifty five patients (or guardians in the Pediatric clinic) participated in the study. Two patients refused to participate. Data of three participants were excluded due to insufficient information. There were 105 (30.5%) males and 240 (69.5%) females. Mean age was 45.8 (SD 17.6) years. There were 140 participants over the age of 50 years and 27 participants less than 20 years.

The participation from each specialist clinic was as follows: 120 for General Medicine (33.8%), 89 for Pediatrics (25.1%), 83 for General Surgery (23.4%) and 63 for Cardiothoracic Surgery (17.7%). Educational status of the participants were as follows: 37.7% never attended school or less than 11 years of school education, 34.5% was educated up to G.C.E. Ordinary Level (Examination after 11 years of school education), 27.8% was educated up to G.C.E Advanced Level (Final examination in school) or had tertiary education.

Of the participants, 172 (48.3%) had heard about the Internet. Awareness about the Internet was dependant on age (p < 0.05 using T-test), educational level (p < 0.05 using Chi-Square) and the clinic they were attending (p < 0.05 using Chi-Square). Higher proportions of participants were aware of the Internet in younger age groups than in older age groups and in higher educational status than with lower educational status. A higher proportion of participants from the Pediatric clinic were aware of the Internet compared to other clinics. A lesser number of participants from Cardiothoracic Surgery clinic were aware of the Internet compared to all the other clinics. Ten participants who 'never attended school' were not aware of the Internet while three out of twelve (25%) participants who had tertiary education were not aware of the Internet. There was no gender difference in awareness of the Internet.

In multivariate analysis, (Table 1) odds of Internet awareness ('No Internet awareness' = 1 and 'Internet awareness' = 0) were significantly low among those who had education up to G.C.E Ordinary Level or less than that and among those who were above 50 years of age. Internet awareness was low among those who were 41 to 50 years of age (p 0.074) and among those who attended the Cardiothoracic clinic (p 0.097) although it did not reach a statistically significant level.

**Table 1:** Logistic regression analysis of the Internet awareness by the educational level, clinic and age ('No internet awareness' = 1 and 'Internet awareness' = 0) (N = 355).

Variable	Categories	Aware	Not aware	Odds R	95% conf. Int.	P value
Education	A' Level & above	71 (74.7%)	24 (25.3%)	1.000		Reference
	Ordinary Level	64 (54.2%)	54 (45.8%)	2.49	1.38 – 4.49	0.002
	< 11-years/no school	32 (31.3%)	97 (68.8%)	8.96	4.86 – 16.52	0.000

Clinics	Paediatric	54 (60.7%)	35 (39.3%)	1.000		Reference
	Medical	55 (45.8%)	65 (54.2%)	1.092	0.52 – 2.25	0.812
	Surgical	40 (48.2%)	43 (51.8%)	1.696	0.84 – 3.40	0.138
	Cardiothoracic	22 (35.5%)	40 (64.5%)	1.975	0.88 – 4.41	0.097

Age	Below 20 years	19 (70.4%)	8 (29.6%)	1.000		Reference
	21–30 years	41 (68.3%)	19 (31.7%)	1.101	0.40 – 2.95	0.849
	31–40 years	36 (58.1%)	26 (41.9%)	1.715	0.65 – 4.51	0.275
	41–50 years	25 (49.0%)	26 (51.0%)	2.470	0.91 – 6.66	0.074
	Over 50 years	46 (32.8%)	94 (67.2%)	4.85	1.97 – 11.91	0.001

Awareness about the Internet were significantly low among those who had education up to G.C.E Ordinary Level or less than that and among those who are above 50 years of age. The Internet awareness was low among those who were 41 to 50 years of age and among those who attended the Cardiothoracic clinic although it did not reach a statistically significant level.

Only three participants (0.97%) have used the Internet to find information on their conditions; one participant attending a clinic in THK and two participants attending clinics in SH. Ages of those who used the Internet were between 20 to 30 years. Close relatives searched the Internet on the conditions of two adult participants. Altogether, information was sought on the Internet only for conditions of five participants. Those who sought information on the Internet mentioned that the information was useful to increase their knowledge on the disease condition. Those five participants whose disease conditions were searched for on the Internet belonged to the following clinics: two parents from the Pediatric clinic, (one had a child with mild short term cough and the other had a child with common cold and cough), one from the General Surgery clinic with swelling in the neck, one from General Medical clinic with Diabetes Mellitus, one from the Cardiothoracic Surgery clinic after a Mitral valve replacement surgery. There was no significant difference in Internet usage or awareness between the participants from the two hospitals. However, there was a difference in educational level between the participants from the two hospitals (p < 0.05 using Chi-Square).

## Discussion

The Internet was used to study the conditions of only 1.4% participants, which is a very low value compared to the findings in the studies in high-income countries. Very high usage of the Internet by those who attended the clinics in developed countries was reported in early studies [2-6]. There were studies about fairly good use of the Internet for health education in low-income countries; Iran (the use of a web-based education system for patients with inflammatory bowel disease which showed fairly good usage)[13], India (successful implementation of Information and Communication Technology based project for local public heath sector which emphasizes the requirement of connectivity, content, capacity building, and policy for bridging the digital divide) [14], Colombia (web-based tele-consulting service to under-served areas to improve the access to health care and information to the community and to encourage open discussion) [15] and China (web-based intervention for improving HIV/AIDS knowledge in rural areas which emphasizes the need for ongoing logistic support for the success of such a project.) [16].

There are several possible reasons for low Internet usage. One possible reason is poor awareness about the Internet, which is a main finding in this study. More than 50% of participants in the current study have not heard about the Internet. This is a very high proportion compared to previous studies in high-income countries. The awareness about the Internet was not even included as an important component in most studies conducted in developed countries; instead their main concern was the availability of the Internet. One health related study in the UK showed that almost everybody (99%) is aware of the Internet [17]. Poor awareness seems to be associated with the age of the patient. Results show that the awareness is low among the participants who are older than 50 years. This has been described previously in high-income countries where elderly people use the Internet less frequently than young people for health information. [18,19] Furthermore, more than half of the participants are over 50 years old in the current study. This may have contributed to the low Internet usage and poor awareness of the Internet in the current study. Those few who used the Internet in the current study are younger than 30 years of age. This is an evidence for the digital divide on age in this study sample, which has been described in previous studies [18,19].

Awareness about the Internet shows a difference according to the clinic they attended. Awareness is low among the participants from the Cardiothoracic Surgery clinic. More participants from this clinic are elderly patients. Therefore, poor awareness among the participants from the Cardiothoracic Surgery clinic can be due to their age. Internet usage as well as the awareness of the Internet shows a correlation with educational level of participants. The majority of participants did not complete G.C.E. Ordinary Level school education in this study. Relatively poor educational level in the country compared to high-income countries has a major effect on Internet usage. Conditions might be worse in most other developing countries where literacy rates are lower than that of Sri Lanka [12]. In African countries, it was described that Internet usage is correlated with the level of education. A study comparing the data from 45 African countries showed that improved school enrolment increases the Internet usage [20]. Since most health information on the Internet is available in English, knowledge of the English language may have an influence on Internet usage for health information retrieval among the participants in the current study. We did not assess participants' English reading ability, which can be considered as a limitation of this study.

The elderly and less educated people in society may not benefit as much from web-based educational programs. Poor awareness about the Internet is one possible reason for this. When improving the Internet as a health education medium, more attention should be paid on these two groups since a considerable proportion of health care receivers belong to these two categories in a country like Sri Lanka.

Another possible factor affecting Internet usage is their disease condition. We recorded the disease conditions of all participants but since there were very few Internet users we were unable to find any relationship between Internet usage and the disease condition or the clinic they were attending.

Low availability of Internet facilities can be the other main reason for lower usage. Unlike in developed countries where most studies were carried out, availability of the Internet is not easy to define in this population. Availability for a family member in his working place, school or even having an Internet-cafe in the village can be considered as availability for most families. This can be exemplified by the fact that out of five users of the Internet in the current study, two users were close relatives of an adult patient. The Internet penetration is low in the country (recorded as 3.7% in 2008 which included 'anyone currently in capacity to use the Internet' [10]). We did not investigate Internet availability in this study instead we questioned about the awareness of the Internet.

Almost all the services are free in the government hospitals contrary to the private hospitals in Sri Lanka. Generally, patients attending the clinics in a government hospital can be considered as a relatively low-income group. In this study, we included patients from both a government and a private hospital since we thought those were two different categories of patients. This study revealed that the participants from the private hospital had a higher educational level, so it is possible for them to have a higher income. Since previous studies have shown a higher Internet usage in high-income and well-educated groups, we expected a difference in Internet usage and awareness in the participants in those two groups although we did not find any such difference [18,19].

Galle is the main city in the Southern Province of Sri Lanka. THK is a tertiary care hospital while SH is one of the main private hospitals in the Southern Province. Socio-economic status in most provinces is lower than that of the Southern Province in Sri Lanka [21]. We cannot expect higher values for Internet usage and awareness generally in Sri Lanka than the values found in this study.

## Conclusion

The very low usage of the Internet for health information retrieval in this study population is probably due to the low awareness of the Internet and the low educational level. This low usage of the Internet and the associated reasons seen in this study can be generalized to Sri Lanka and probably to other low income countries which have lower educational levels than Sri Lanka.

## Competing interests

The author declares that they have no competing interests.

## Authors' contributions

Author designed the study, analyzed data and wrote the manuscript and contributed in data collection.

## Pre-publication history

The pre-publication history for this paper can be accessed here:

http://www.biomedcentral.com/1472-6947/9/12/prepub
